# Imaging Findings of Metaplastic Carcinoma of the Breast with Pathologic Correlation

**DOI:** 10.5334/jbsr.1386

**Published:** 2018-04-27

**Authors:** Henrique Donato, Isabel Candelária, Pedro Oliveira, Manuela Gonçalo, Filipe Caseiro-Alves

**Affiliations:** 1 University Centre Hospitals of Coimbra (CHUC), PT

**Keywords:** metaplastic carcinoma of the breast, breast neoplasms, mammography, breast ultrasonography, magnetic resonance imaging

## Abstract

**Objectives:**

To review the imaging findings of a series of cases of metaplastic carcinoma of the breast, a rare and aggressive form of breast cancer with variable imaging features.

**Materials and methods:**

Retrospective review of multimodality imaging features of eleven cases of metaplastic carcinoma of the breast retrieved from a single hospital institution database. Clinical and pathologic data were also documented.

**Results:**

The median age of presentation was 65 years. Four cases had axillary lymphadenopathies, and two had distant metastases. An oval mass was the most common sonographic finding (7/11; 64%). Lesions displayed circumscribed/partially circumscribed margins (6/11; 55%) or non-circumscribed margins (5/11; 45%). Most lesions had a heterogeneous echo structure (9/11; 82%) and posterior acoustic enhancement (6/11; 55%). In nine patients, mammographies were available. An oval dense mass was the most common mammographic finding (5/9; 56%). The majority of cases had non-circumscribed margins (6/9; 67%), and nearly half displayed calcifications (4/9; 44%).

**Conclusions:**

Mammographic findings were not different from the usual features of more prevalent types of breast cancer, though the majority of metaplastic carcinoma of the breast showed possible distinctive sonographic features, such as circumscribed margins or complex echogenicity, reflecting the histologic background.

## Introduction

Metaplastic carcinoma of the breast (MCB) is a rare and aggressive form of breast cancer, representing less than 1% to 5% of cases [1,2,3,4]. It should be distinguished from invasive ductal carcinoma, because MCB has a worse prognosis, and from breast sarcoma, because the treatment approach is different [5,6].

Most of the cases of MCB are sporadic, with unknown etiology [3,7]. In fact, MCB is a general term applied to a heterogeneous group of breast cancers, which have in common a metaplastic change to a non-glandular component [1,8,9].

The type and degree of metaplastic change is variable, leading to different histopathologic subtypes [10,11,12]. This has resulted in multiple designations and various classification systems, such as the World Health Organization (WHO) classification and the Wargotz and Norris classification [1,11].

While MCB subtypes with different epithelial populations can be diagnosed solely by the morphological aspects at pathology, subtypes with a mesenchymal component need immunohistochemistry analysis to differentiate from breast sarcoma. The differentiation is made by detecting the coexpression of markers of mesenchymal (vimentin) and epithelial cells (cytokeratin) [3,13,14].

These highly variable histopathologic findings lead to variable and, ultimately, non-specific imaging features, the assessment of which is further complicated by the rarity of the disease [5,6,7,11]. MCB has been predominantly reported as displaying more benign characteristics than invasive ductal carcinoma [1,7]. However, other studies did not find such distinction [6,13,14,15].

The purpose of this study is to review the imaging findings of a series of cases of MCB comprising different modalities and also to assess clinical and pathologic findings.

## Materials and Methods

Eleven histologically proven cases of MCB were retrieved from the pathology department database of a single hospital institution. These cases were diagnosed between January 2006 and December 2016. Additional histologic data (histologic subtype according to Wargotz and Norris classification, histologic grade, lesion receptor status and presence of lymphadenopathies) and clinical information (age and symptoms at presentation) were obtained from the hospital records.

Two experienced radiologists performed a retrospective consensus review of the pre-operative breast imaging studies or imaging reports in three exams where the images were not available. Imaging studies included mammography, breast ultrasound and breast magnetic resonance (MR). All reports used the BI-RADS lexicon.

Regarding the assessment of mammographic findings, lesions were classified as a breast mass or asymmetry. The shape of the lesion (oval, round, irregular), margins (circumscribed, partially circumscribed, non-circumscribed), density (low, equal, high), presence of calcifications and their appearance (suspicious, not suspicious) and associated features (skin thickening, skin retraction) were also registered.

Regarding sonographic findings, lesions were classified according to shape (oval, round, irregular), orientation (parallel, non-parallel), margins (circumscribed, partially circumscribed, non-circumscribed), echo structure (cystic, complex solid and cystic, solid hypoechoic, solid isoechoic, solid hyperechoic) and posterior acoustic features (enhancement, shadowing, none).

Lesion size and presence of lymphadenopathies at imaging exams were also assessed.

Regarding MR features, T1 and T2 signal intensity (hyperintensity, isointensity, hypointensity), signs of pectoris major invasion and dynamic assessment (enhancement pattern and curve type) were also registered.

The median values of patient age at presentation and lesion size were determined.

## Results

### Clinical and pathologic findings

The median age at presentation was 65 years (ranging from 30–86 years), and the most frequent presenting symptom was a palpable breast nodule, which was observed in five of the eleven patients (45%). Four patients (36%) were asymptomatic, and the lesion was discovered on breast screening.

Applying the Wargotz and Norris classification, there were three cases of squamous subtype, three cases of carcinosarcoma and three cases of matrix producing MCB. There were also two cases of spindle cell subtype, one of them was determined to be a fibromatosis-like variant (Tables 1 and 2).

**Table 1:** Clinical and pathologic findings of 11 cases of metaplastic carcinoma of the breast with histologic subtype classification.

Case	Histologic subtype	Hormonal receptors	HER2 mutation	Axillary lymphadenopathies	Distant metastases

**1**	Squamous cel	**+**	**+**	**–**	**–**
**2**	Carcino-sarcoma	**–**	**–**	**+**	**–**
**3**	Spindle cell	**–**	**–**	**–**	**–**
**4**	Matrix producing	**–**	**–**	**+**	**+**
**5**	Carcino-sarcoma	**–**	**–**	**–**	**–**
**6**	Spindle cell fibromatosis-like variant	**–**	**–**	**–**	**–**
**7**	Squamous cell	**–**	**–**	**+**	**–**
**8**	Squamous cell	**+**	**–**	**–**	**–**
**9**	Carcino-sarcoma	**–**	**–**	**–**	**–**
**10**	Matrix producing	**–**	**–**	**–**	**–**
**11**	Matrix producing	**–**	**–**	**+**	**+**

*Note:* + positive/present; – negative/absent.

**Table 2:** Mammographic and sonographic findings of 11 cases of metaplastic carcinoma of the breast with histologic subtype classification.

Case	Histologic subtype	Mammographic findings			Sonographic findings			

		Shape	Margins	Calcification	Mass shape	Margins	Echo structure	Posterior acoustic findings

**1**	Squamous cell	Oval mass	Non-circumscribed	+ (suspicious)	Irregular	Non-circumscribed	Complex	Enhancement
**2**	Carcino-sarcoma	Oval mass	Non-circumscribed	+ (suspicious)	Irregular	Non-circumscribed	Complex	None
**3**	Spindle cell	Oval mass	Partially circumscribed	–	Oval	Non-circumscribed	Solid hypo-echoic	Shadowing
**4**	Matrix producing	Global asymmetry	Non-circumscribed	+ (suspicious)	Irregular	Non-circumscribed	Complex	Shadowing
**5**	Carcino-sarcoma	Oval mass	Non-circumscribed	+ (suspicious)	Oval	Circumscribed	Complex	Enhancement
**6**	Spindle cell fibromatosis-like variant	Round mass	Circumscribed	–	Oval	Circumscribed	Solid hypo-echoic	None
**7**	Squamous cell	Irregular mass	Non-circumscribed	–	Oval	Partially circumscribed	Complex	Enhancement
**8**	Squamous cell	NA	NA	NA	Oval	Circumscribed	Complex	Enhancement
**9**	Carcino-sarcoma	NA	NA	NA	Oval	Partially circumscribed	Complex	Enhancement
**10**	Matrix producing	Oval mass	Partially circumscribed	–	Oval	Partially circumscribed	Complex	Enhancement
**11**	Matrix producing	Focal asymmetry	Non-circumscribed	–	Irregular	Non-circumscribed	Complex	None

*Note:* NA – not available.

Nine patients (82%) did not express hormonal receptors, and ten (91%) were negative for HER2 mutation. On histologic analysis, nine lesions (82%) were high-grade G3.

Four patients had histologically confirmed axillary lymphadenopathies (36%) at presentation, and two patients developed distant metastases (Table 1).

### Imaging findings

The median size determined by the imaging studies at diagnosis was 27 mm (with a range of 11–100 mm).

Of the four patients with axillary lymphadenopathies, two were identified by imaging. One of the missed cases had only one positive axillary lymph node out of 24.

Ultrasound was performed in all patients (Table 2). Seven cases presented as oval masses, which was the most common ultrasound finding. Five of these lesions had a parallel orientation, and two had a non-parallel ‘taller than wide’ suspicious appearance.

The majority of lesions displayed circumscribed/partially circumscribed margins (55%) at sonographic assessment.

**Figure 1:**
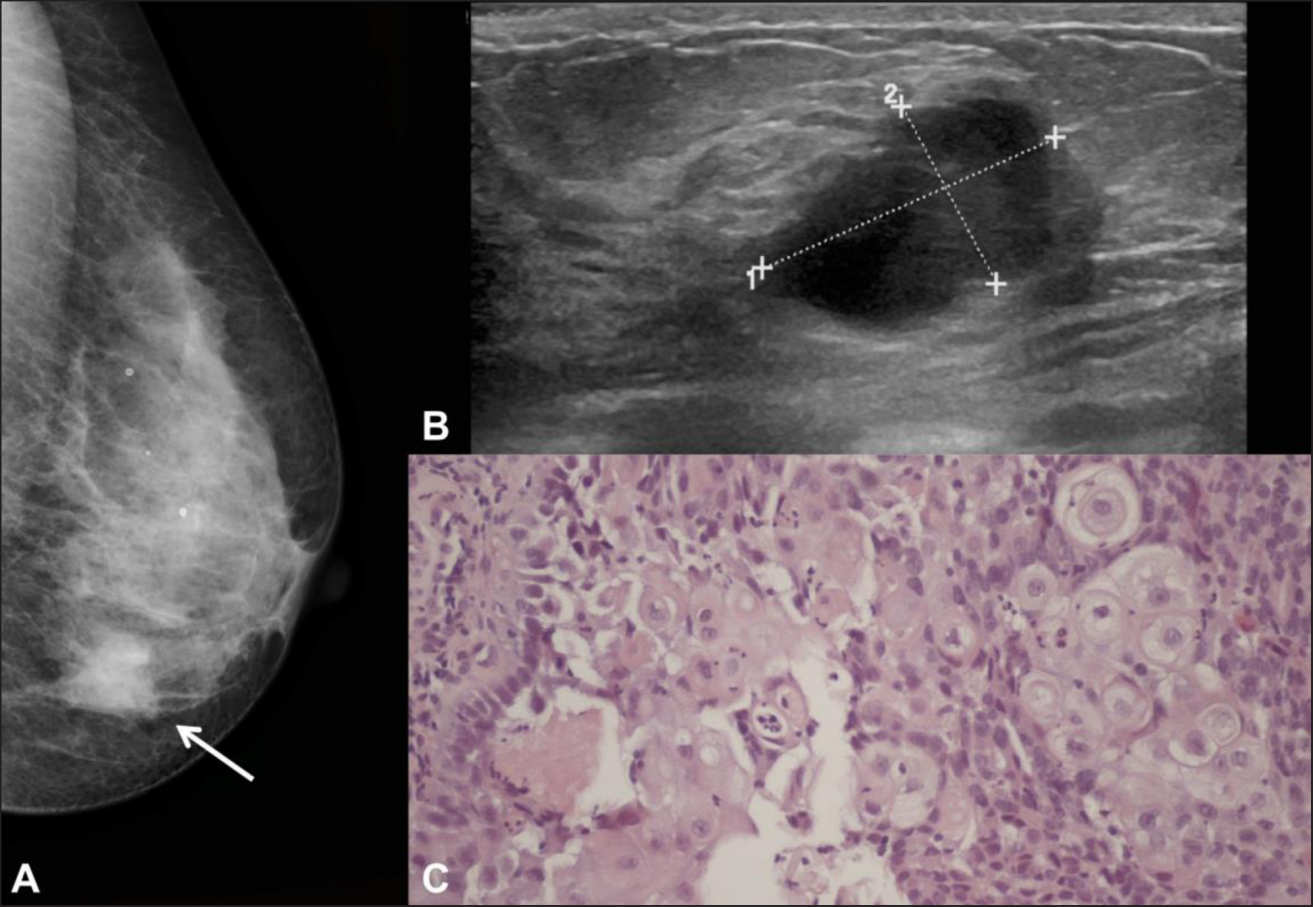
Example of squamous cell subtype in a 66-year-old woman. **A** – Left craniocaudal mammogram showing an irregular dense mass in the inner quadrants of the breast (arrow), with non-circumscribed margins. **B** – At ultrasound, it corresponded to a partially circumscribed breast mass with complex solid and cystic echo structure and posterior acoustic enhancement. **C** – Photomicrograph showing squamous cell differentiation (H&E, 200x).

**Figure 2:**
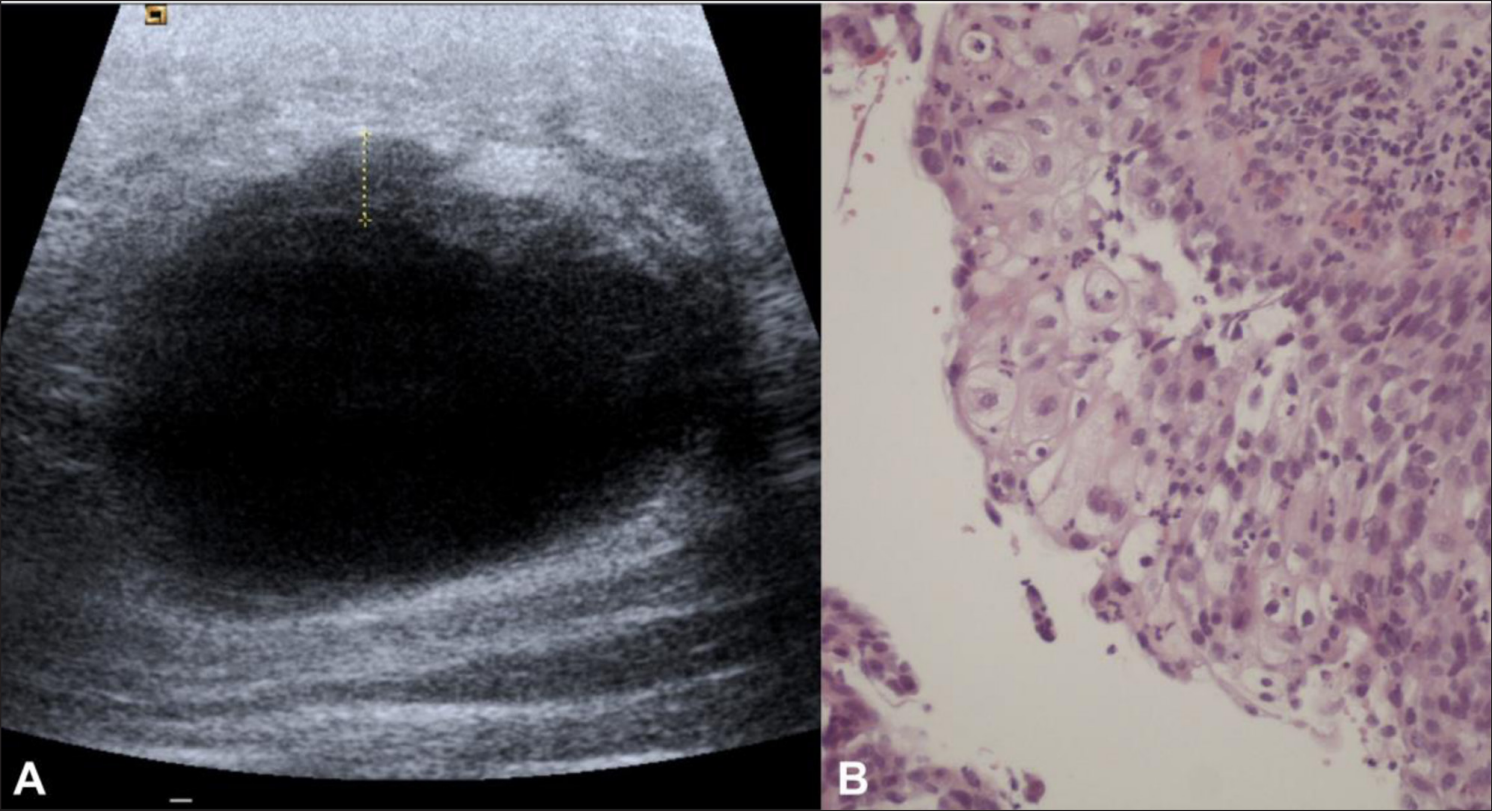
Large left breast mass in a 36-year-old woman with squamous cell subtype. **A** – Sonogram demonstrating a large cystic lesion with irregular anterior wall thickening. **B** – Photomicrograph showing squamous cell differentiation (H&E, 200x).

Nine lesions (82%) had a heterogeneous echo structure, with complex solid and cystic components belonging to squamous cell, matrix producing and carcinosarcoma subtypes (Figure 1). In two lesions, the cystic component was predominant (Figure 2). The remaining two lesions were solid hypoechoic at ultrasound, corresponding to the two cases of spindle cell subtype. Six lesions (55%) had posterior acoustic enhancement.

**Figure 3:**
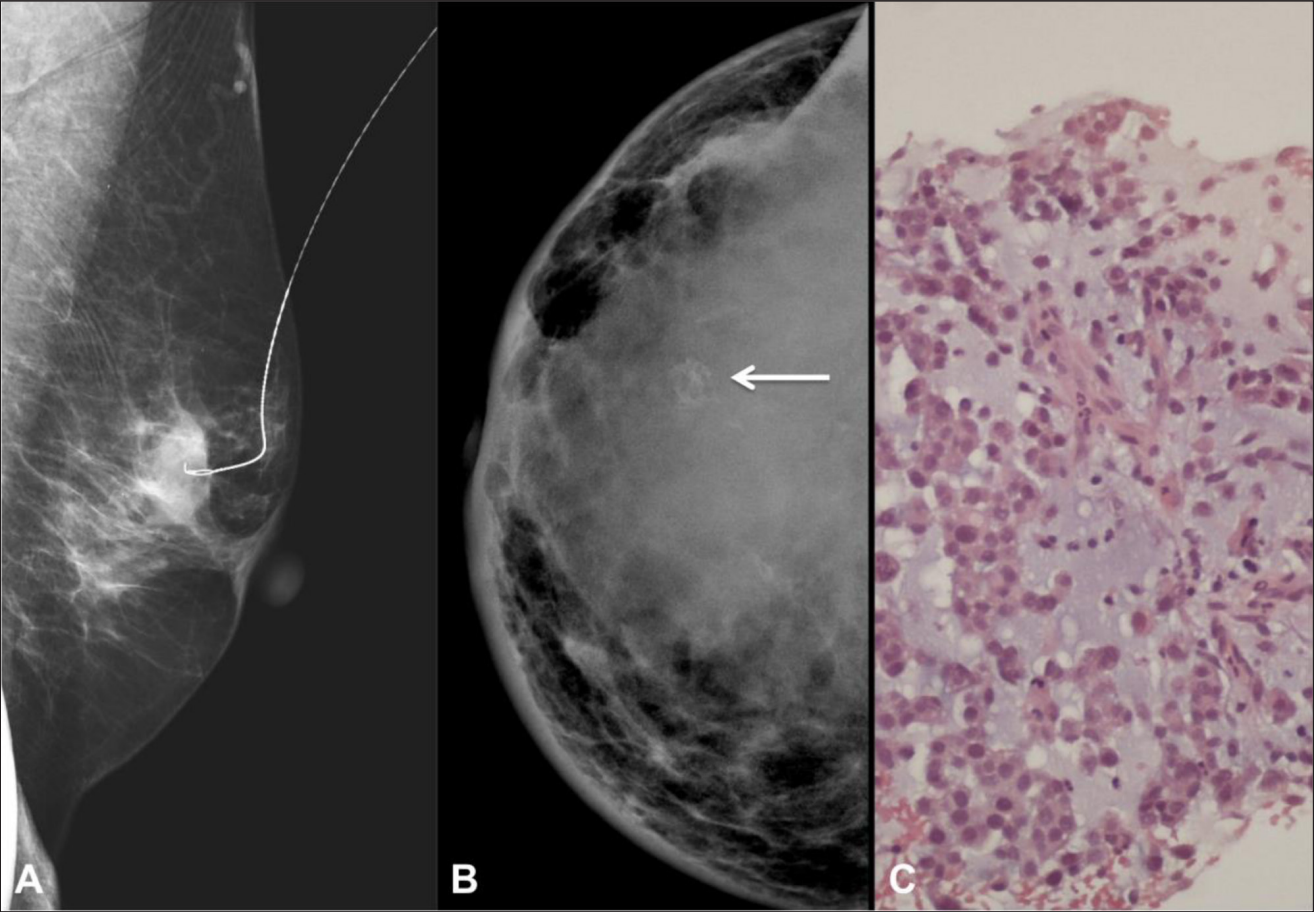
**A** – Left mediolateral oblique mammogram of a 68-year-old woman with spindle cell subtype. Pre-operative needle for localization in a partially circumscribed oval dense mass. **B** – Right craniocaudal mammogram of a 30-year-old woman with matrix producing subtype, showing a global asymetry with coarse calcifications (arrow). There is also associated skin thickening (especially in the inner quadrants) and nipple retraction. **C** – Photomicrograph of the same lesion in B demonstrating the presence of chondromyxoid matrix (H&E, 200x).

Mammography was performed in nine of the eleven patients (Table 2). The most common mammographic finding was an oval dense mass, present in five out of the nine patients (56%) (Figure 3). There were two cases that presented as breast asymmetries.

The majority of cases had non-circumscribed margins (67%) (Figure 1).

Four lesions had associated calcifications (44%), all of suspicious morphology. Three of them were pleomorphic, and the remainder case had coarse heterogeneous calcifications, corresponding to a case of matrix producing MCB (Figure 3).

Only one case showed associated skin thickening and retraction (Figure 3).

**Figure 4:**
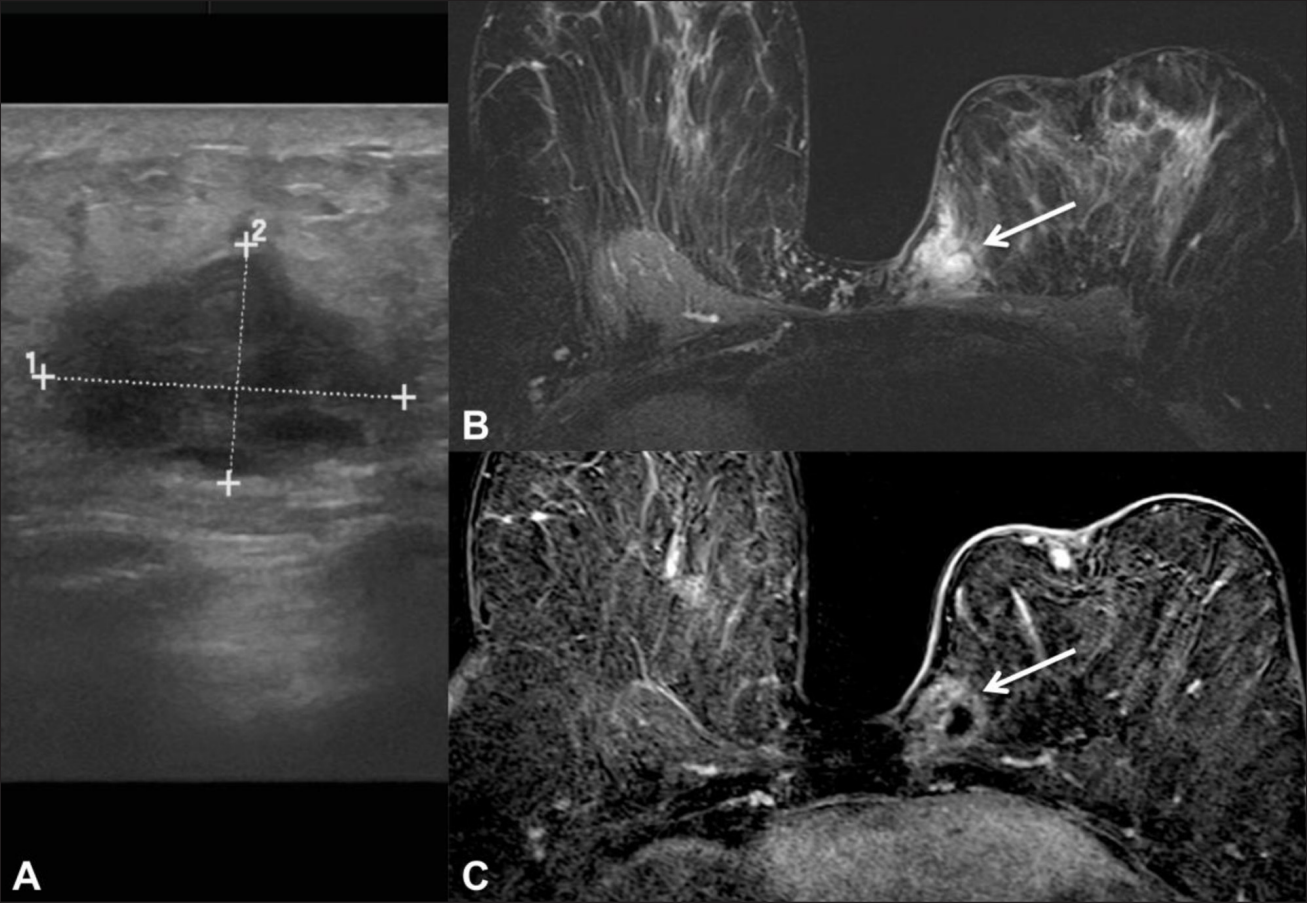
62-year-old woman with carcinosarcoma subtype. **A** – An oval mass with complex solid and cystic echo structure and posterior acoustic enhancement was detected in the inner quadrants of the left breast at ultrasound. **B** – Breast MR T2-W fat suppression acquisition showing a corresponding irregular mass with areas of marked T2 hyperintensity (arrow). **C** – Subtracted T1-W fat suppression image after intravenous paramagnetic contrast, demonstrating ring-like enhancement (arrow) and signs of pectoris major muscle invasion.

Breast MR was only performed in two advanced cases. In both of them, this imaging modality revealed T1 hypointense and heterogeneous T2 hyperintense masses, with areas of marked T2 hyperintensity. Dynamic analysis revealed heterogeneous enhancement in one case with a type III curve and peripheral enhancement with a non-suspicious type I curve in the other case (Figure 4). In both cases, there was invasion of the pectoris major muscle.

## Discussion

### Clinical and pathologic findings

The most frequent clinical finding of MCB is a palpable breast mass, similar to the more common forms of breast cancer [1,2,4]. However, MCB have been described as presenting at a younger age, with larger tumor size and a more rapid growth than the more prevalent forms of breast cancer [1,2,8,10]. Nevertheless, some authors have found that MCB can be more common in women older than 50 years, such as in this series that has a median age of 65 years at presentation [4,13].

Lymph node involvement by MCB is less frequent than in the most prevalent forms of breast cancer, especially regarding the relative large size of the lesions [1,7,10]. The incidence of 36% determined in this study is within the 25%–40% range reported in the literature [2,13,16]. However, lymph node involvement is definitely more common than in breast sarcoma, leading to a different treatment approach, because axillary dissection is not generally performed in the latter [5,8,14].

Hematogenous spread is reported as more frequent than lymphatic spread in MCB, reflecting a more sarcomatoid behavior. Lung and bone are the most common metastatic sites [1,2,10]. Only two cases showed distant metastatic spread in this series, probably due to an early diagnosis performed at screening in more than a third of cases.

There are various pathologic classification systems for MCB. The main classifications are the WHO classification, with a recent update, and the more popular Wargotz and Norris classification, used in this study (Table 3) [1,9,11,15,17].

**Table 3:** Pathologic classification systems for Metaplastic Carcinoma of the Breast.

Updated WHO Classification	Wargotz and Norris Classification

– Squamous cell carcinoma	– Spindle cell subtype
– Spindle cell carcinoma	– Squamous cell carcinoma subtype
– Low grade adenosquamous carcinoma	– Carcinosarcoma
– Metaplastic carcinoma with mesenchymal differentiation	– Mixed metaplastic carcinoma
– Fibromatosis-like metaplastic carcinoma	– Osteoclastic giant cell subtype
– Matrix producing subtype	

WHO – World Health Organization.

The spindle cell subtype of MCB is difficult to differentiate from low grade sarcoma and granulation tissue [1]. A low grade fibromatosis-like variant has been described, warranting distinction due to its favorable prognosis and being already included as a subtype in the updated WHO classification [2,11,15].

The squamous cell carcinoma subtype has malignant squamous cells independent of the overlying skin and other primary sites. The differential diagnosis therefore includes metastasis to the breast and also phyllodes tumor [18].

The carcinosarcoma subtype represents very aggressive tumors, with clearly malignant epithelial and mesenchymal components [1,9].

The matrix-producing subtype corresponds to invasive carcinomas with areas of chondroid or osseous differentiation [19].

Finally, the osteoclastic giant cell subtype is a carcinoma with spindle cell or sarcomatous matrix plus osteoclastic cells [1].

MCB generally has an aggressive behavior with higher proliferation index and poorer differentiation than the more common forms of breast carcinoma. As seen in this study, it usually presents as a triple negative or basal-like breast cancer, being less associated to expression of estrogen, progesterone and HER2 receptors. These characteristics further reduce the outcome of MCB patients by limiting treatment options [1,3,4,10].

### Imaging findings

#### Ultrasound

MCB has a variable appearance on ultrasound [11,18]. The lesions can be oval, round, lobular and, less frequently, irregular, and the margins may be circumscribed or non-circumscribed. However, MCB has generally been described as having a more benign appearance than invasive ductal carcinoma [1,2]. This was also observed in this series, with most lesions presenting as an oval mass with either circumscribed or partially circumscribed margins.

MCB can present as a solid breast mass but more frequently displays a complex echo structure with cystic components. These cystic areas correspond to necrosis, hemorrhage or cystic degeneration on histology [1,8,13,16]. Possibly due to the cystic component or to lesion hypercellularity, there is frequent posterior acoustic enhancement, creating greater confusion in the distinction of benign lesions [2,5,8,16]. This is in clear contrast to invasive ductal carcinoma, generally displaying posterior acoustic shadowing [1]. Complex solid and cystic breast lesions may represent malignant entities and, although rare, MCB should be included in the differential diagnosis [6].

#### Mammography

A dense mass is the most frequent feature of MCB on mammography [1]. Margins vary from circumscribed to partially circumscribed to non-circumscribed, the most common type in this series [1,7]. MCB have been more commonly described as having a generally more benign appearance than invasive ductal carcinoma, with round, lobular or oval shape and predominantly circumscribed margins [1,7,13,20]. However, as in this series, there are studies, including a recent larger series, that describe MCB as a mass similar to invasive ductal carcinoma on mammography, with no classic or distinctive feature regarding margin [4,6,13,15,18]. A reason for the non-circumscribed margins is the presence of an invasive ductal carcinoma component [8].

Calcifications are often absent in MCB and are described in up to 25% of cases. If present, they can be amorphous, coarse, punctate or pleomorphic [1,8,13]. While the majority of lesions in this series did not display calcifications, the rate of 44% is higher than the one reported. Calcifications are more frequent in matrix producing subtypes, such as carcinomas with chondroid metaplasia, with an example shown in this series. Ossification, when present, is also evident on mammography [5,9,13].

Associated features such as architectural distortion, is uncommon in MCB [13].

It has been mentioned that, despite its rarity, MCB should be considered in the differential diagnosis of a predominantly circumscribed high-density breast mass without calcifications. The presence of partially circumscribed margins is a possible distinctive feature [6,7].

#### Combined imaging findings

The combined mammographic and sonographic features of MCB may be misinterpreted as a BI-RADS 3 lesion, creating difficulties and delaying diagnosis [2,12]. The more benign appearance of this type of tumor is in contradistinction to its greater aggressiveness and worse prognosis [8]. On this series, the majority of lesions had a combined malignant appearance. There were, however, sonographic features less frequently associated to the more prevalent types of breast cancer, such as circumscribed or partially circumscribed margins, complex echogenicity with cystic components and posterior acoustic enhancement [1,4].

#### Magnetic resonance

Like on other imaging modalities, MCB is more frequently described as a round or lobular mass with relatively smooth margins, although it may also present as an irregular mass with spiculated margins [1,12]. Similar to other types of breast cancer, MCB is T1 iso- or hypointense [1,13]. On the other hand, T2 sequences are important for the differential diagnosis, because MCB is generally heterogeneously T2 hyperintense [1,8,13]. There are T2 iso- or hypointense solid components and hyperintense portions, frequently attributed to necrosis, but also to cystic degeneration, myxoid matrix, intratumoral hemorrhagic changes and loose edematous stroma [1,5,12,13,21]. For the same reasons, the enhancement pattern is frequently heterogeneous or ring-like [1,5,8]. Dynamic contrast analysis may render suspicious type II or III curves, but also benign type I curves [5,8,13].

### Radiologic-Pathologic correlation

MCB frequently presents with central necrosis, reflected in the complex appearance and cystic components at ultrasound, the characteristic T2 hyperintensity and ring-like enhancement after contrast at breast MRI [13]. Lesions with cystic areas are generally attributed to a squamous component [1,6]. In this series, only the cases belonging to the spindle cell subtype did not show complex echogenicity.

The spindle cell component has generally more circumscribed margins, while more irregular margins are associated to an invasive epithelial component [1,8,11].

The presence of partially circumscribed margins, frequently described in these tumors and a possible distinctive feature, reflects the existence of both the metaplastic component and the invasive carcinoma component [6,7].

## Limitations

The reduced number of assessed cases is the main limitation of this study and may explain the variable imaging findings. Unfortunately, this is also true for many published studies, where there is a small series due to the rarity of the disease. Analysis of MCB subtypes is even more limited. The literature is not unanimous regarding MCB imaging findings, with many authors referring more benign features, while others report features similar to the more prevalent forms of breast cancer [1,4,6,13,14,15].

Studies with larger series are needed to better characterize the imaging features of this heterogeneous group of breast tumors.

The retrospective nature of the study is another limitation. In three imaging studies, only the imaging report was available for review. Additionally, while mammographic and MR findings can be readily interpreted, sonographic findings are dependent on the performance and the detection of imaging features at the time of the exam. However, all imaging studies had been reported according to the BI-RADS lexicon.

## Conclusion

MCB has a variable appearance on imaging studies. Mammographic findings were not different from the usual features of more prevalent types of breast cancer. However, on ultrasound, the majority of cases showed possible distinctive features, such as circumscribed/partially circumscribed margins, complex echogenicity and posterior acoustic enhancement, reflecting the histologic background of these tumors. Although rare, inclusion of MCB in the differential diagnosis of these features may be warranted.
